# Exogenous ketosis in breath‐hold diving: Promise, paradox and physiological risk

**DOI:** 10.1113/EP094163

**Published:** 2026-10-08

**Authors:** Ivan Drviš, Myrthe Stalmans, Dario Vrdoljak, Nikola Foretic, Damian M. Bailey, Željko Dujić

**Affiliations:** ^1^ Faculty of Kinesiology University of Zagreb Zagreb Croatia; ^2^ Department of Cellular and Physiological Sciences, Faculty of Medicine University of British Columbia Kelowna British Columbia Canada; ^3^ Centre for Heart, Lung and Vascular Health University of British Columbia, Okanagan Campus Kelowna British Columbia Canada; ^4^ Faculty of Kinesiology University of Split Split Croatia; ^5^ Neurovascular Research Laboratory, Faculty of Life Sciences and Education University of South Wales Pontypridd UK; ^6^ Department of Integrative Physiology University of Split School of Medicine Split Croatia

**Keywords:** apnoea, aquaticity, breath‐hold diving, carbon dioxide, cerebral blood flow, chemoreflex, ketone ester

## Abstract

Exogenous ketosis induced by ketone ester (KE) ingestion has attracted growing interest as a potential strategy to enhance physiological tolerance to reduced O_2_ availability. Reported benefits arise largely from KE‐induced hyperventilation, which can improve systemic and tissue oxygenation during altitude exposure and hypoxic exercise, potentially complemented by favourable effects on cellular bioenergetics. Whether these responses translate to breath‐hold diving, however, remains unknown. Breath‐hold performance is constrained by the integrated effects of progressive hypoxaemia, CO_2_ accumulation, acid–base disturbance, altered cerebrovascular regulation and chemoreflex‐mediated respiratory drive. Because ventilation ceases during apnoea, interventions that alter pre‐apnoeic acid–base status or CO_2_ signalling might modify the chemical, neural and perceptual stimuli underlying the urge to breathe without slowing O_2_ depletion. Safety therefore depends not only on preserving cerebral O_2_ delivery, but also on maintaining the chemoreflex and perceptual warning signals that ordinarily precede hypoxic loss of consciousness. This review evaluates whether the established physiological effects of KE are mechanistically compatible with the unique demands of apnoea, focusing on acid–base regulation, CO_2_‐dependent cerebrovascular control, autonomic integration and training‐related adaptations in elite freedivers, including reduced chemoreflex sensitivity and enhanced cortical control of respiratory drive. We propose that benefits observed during maintained ventilation might not translate to breath‐hold diving and could create a mechanistic mismatch with uncertain consequences for performance and safety. Direct apnoea‐specific studies are required to determine whether KE supplementation is beneficial, neutral or harmful.

## Abstract (highlights)

- **What is the topic of this review?**
  This review evaluates whether ketone ester supplementation is physiologically compatible with breath‐hold diving, focusing on acid–base regulation, chemoreflex control, cerebral O_2_ delivery and bioenergetics, autonomic integration and safety.
- **What advances does it highlight?**
  Benefits observed during breathing hypoxia might not translate to apnoea, when ventilation is absent. Ketone esters might alter acid–base status, cerebral perfusion and physiological warning signals preceding hypoxic loss of consciousness without slowing O_2_ depletion, revealing a potential mechanistic mismatch with implications for performance and safety.

## 1 CONTEXT

Breath‐hold (BH) diving imposes progressive hypoxaemia and tissue hypoxia in conditions fundamentally different from environmental hypoxia. Once apnoea begins, ventilation ceases, CO_2_ accumulates, O_2_ availability declines and the internal milieu progressively acidifies (Dujic & Breskovic, 2012; Ferretti, 2001; Lindholm & Lundgren, 2009). Performance therefore depends on the integrated regulation of finite O_2_ stores, CO_2_ accumulation, cerebrovascular regulation, chemoreflex signalling, cerebral oxygen delivery (CDO_2_), perception and behavioural control. A defining feature is the dissociation between CO_2_‐driven respiratory discomfort and the ultimate limit imposed by severe hypoxaemia and cerebral deoxygenation (Ainslie & Duffin, 2009). The urge to breathe ordinarily precedes hypoxic loss of consciousness, providing a functional safety margin between behavioural warning and physiological limit. Through training, elite freedivers increase tolerance to hypercapnia and enhance cortical modulation of respiratory drive, allowing apnoea to be prolonged while progressively narrowing this safety margin. Any intervention that modifies acid–base balance, carbon dioxide signalling, oxygenation or respiratory perception must therefore be assessed not only for its ergogenic potential, but also for its effects on the physiological coupling between behavioural warning and the ultimate hypoxic limit.

Against this background, exogenous ketosis has emerged as a potential strategy to support physiological function when O_2_ availability is limited. It is characterised by an acute increase in circulating ketone bodies, principally β‐hydroxybutyrate (βHB) and acetoacetate (AcAc), following ketone supplementation. Although ketone salts and diesters can also induce ketosis, ketone monoesters (KE) are widely used because they rapidly increase circulating βHB, typically within 30 min, and are generally well tolerated (Robberechts & Poffé, 2024). Importantly, KE ingestion induces a transient metabolic acidosis, whereas other ketone formulations can exert different acid–base effects, including mild alkalosis (Stubbs et al., 2017). This acidosis appears to contribute importantly to the responses observed during hypoxic exposure by stimulating ventilation and increasing systemic, skeletal muscle and cerebral oxygenation at rest and during exercise (Stalmans et al., 2024; Stalmans, Tominec, Lauriks et al., 2025). Potential benefits might also extend beyond ventilation: ketone bodies can serve as oxidative substrates (Chegodaev et al., 2022; Cox et al., 2016), can enhance mitochondrial energy transduction (Veech et al., 2001; Sato et al., 1995) and potentially support cerebral bioenergetics during hypoxic stress (Morris, 2005; Rourke et al., 2026; Stalmans et al., 2024; Vermaerke et al., 2026). Whether these potential benefits remain physiologically relevant when ventilation is absent represents the central question addressed in this review.

These findings provide a compelling rationale for studying exogenous ketosis during hypoxia, yet their relevance to BH diving remains uncertain. Reported benefits of KE have been demonstrated almost exclusively while ventilation is maintained and can compensate for metabolic acidosis. During apnoea, this mechanism is unavailable, whereas KE‐induced changes in acid–base status, bicarbonate reserve, CO_2_ signalling and cerebral perfusion might persist. Their physiological consequences might therefore differ fundamentally from those observed during breathing hypoxia because the primary compensatory mechanism, ventilation, is no longer available. Throughout this review, we therefore distinguish between physiological effects demonstrated experimentally during maintained ventilation, mechanisms supported indirectly by related human studies, and apnoea‐specific consequences that remain hypothesis generating. This review critically evaluates whether the established actions of KE are compatible with the unique demands of BH diving, focusing on acid–base regulation, chemoreflex and perceptual control, cerebrovascular and autonomic integration, and implications for performance and safety. The divergent physiological effects of KE during breathing hypoxia and apnoea, together with their principal mechanistic consequences and modifying factors, are summarised in Figure 1.

**FIGURE 1:**
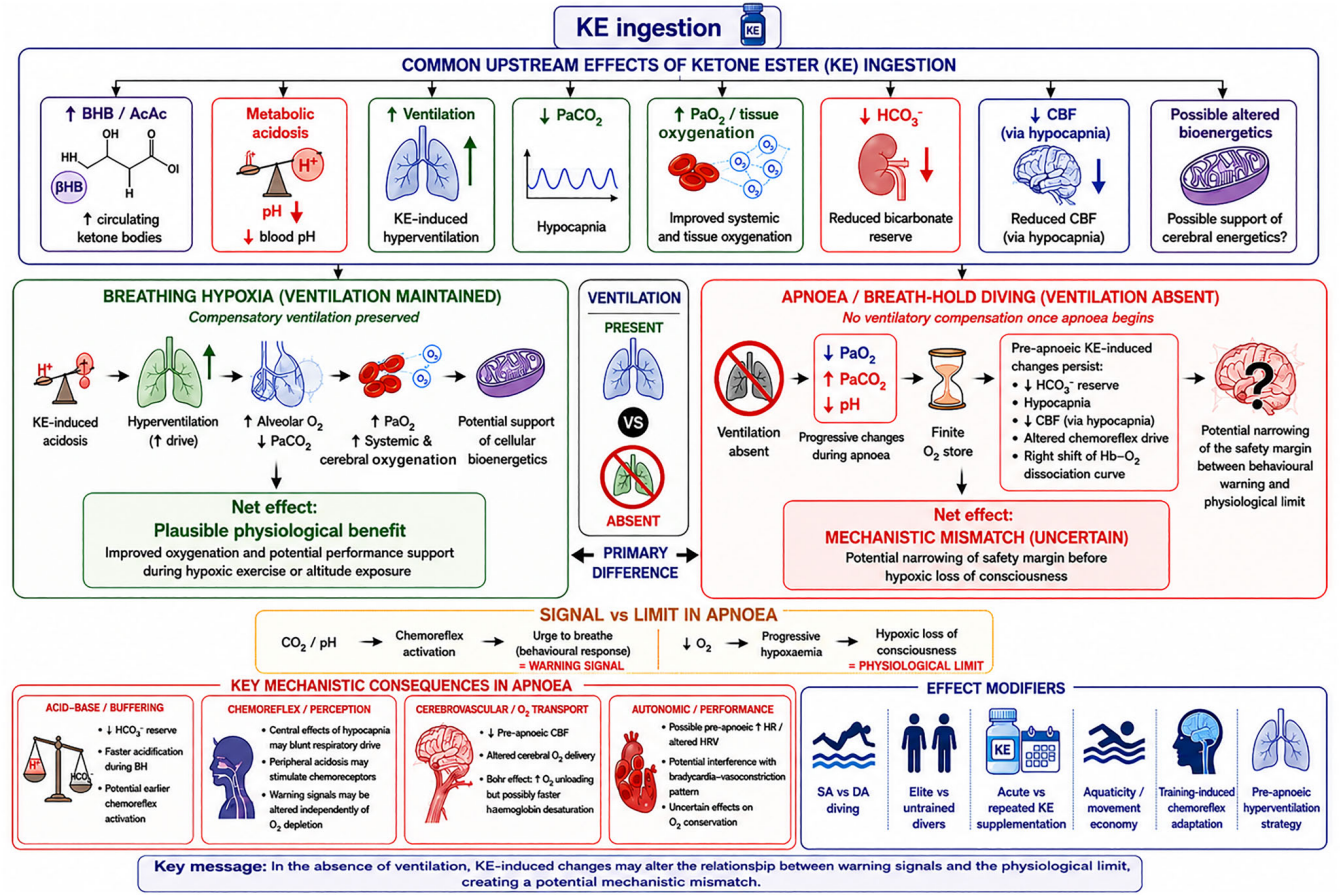
Mechanistic divergence of KE supplementation during breathing hypoxia and breath‐hold apnoea. KE ingestion acutely increases circulating βHB and AcAc, induces metabolic acidosis, stimulates ventilation, lowers $P_{\mathrm{aC}O_{2}}$, increases $P_{aO_{2}}$ and tissue oxygenation, reduces circulating HCO_3_^−^ and may decrease CBF through hypocapnia. KE may also exert direct bioenergetic effects. During breathing hypoxia, preserved ventilation allows KE‐induced hyperventilation to raise alveolar O_2_ availability and $P_{aO_{2}}$, potentially improving systemic and cerebral oxygenation and supporting cellular bioenergetics. During BH apnoea, however, ventilation is absent and $P_{aO_{2}}$ progressively falls while $P_{\mathrm{aC}O_{2}}$ rises and pH declines. Pre‐apnoeic KE‐induced hypocapnia, reduced HCO_3_^−^ reserve, lower CBF, altered chemoreflex drive and a rightward shift of the Hb–O_2_ dissociation curve may therefore persist without continued ventilatory compensation. The resulting effects on O_2_ delivery, extraction, respiratory discomfort and the warning signals preceding hypoxic loss of consciousness remain uncertain. These responses are likely to be modified by static versus dynamic apnoea, training status, acute versus repeated KE supplementation, aquaticity and training‐induced chemoreflex adaptation. Abbreviations: βHB, β‐hydroxybutyrate; AcAc, acetoacetate; BH, breath‐hold; CBF, cerebral blood flow; DA, dynamic apnoea; Hb, haemoglobin; HR, heart rate; HRV, heart‐rate variability; KE, ketone ester; $P_{\mathrm{aC}O_{2}}$, arterial partial pressure of carbon dioxide; $P_{aO_{2}}$, arterial partial pressure of oxygen; SA, static apnoea.

## 2 BREATH‐HOLD DIVING IS NOT ALTITUDE: A FUNDAMENTAL PHYSIOLOGICAL NON‐EQUIVALENCE

BH diving and continuously breathing hypoxia, including high‐altitude exposure, are both O_2_‐limited environments, but their compensatory mechanisms and performance constraints differ fundamentally. This distinction is crucial when extrapolating the effects of interventions such as KE supplementation.

During high‐altitude or normobaric hypoxia, exercise performance and hypoxic tolerance are constrained primarily by reductions in arterial oxygen tension ($P_{aO_{2}}$), while ventilation remains continuously available as the principal compensatory mechanism. The primary compensatory response is hypoxic hyperventilation (Flück et al., 2015; Vrdoljak et al., 2026; Willie, Smith et al., 2014), which raises alveolar $P_{O_{2}}$ and $P_{aO_{2}}$ while lowering arterial carbon dioxide tension ($P_{\mathrm{aC}O_{2}}$), leading to respiratory alkalosis (Ainslie & Subudhi, 2014). Ventilation therefore remains the primary homeostatic regulator of both oxygen and carbon dioxide, continuously reshaping the internal physiological environment throughout hypoxic exposure. Interventions that alter acid–base balance, metabolism or substrate utilisation may consequently interact with this ventilatory response to influence tissue oxygenation and, ultimately, hypoxic tolerance.

Apnoea fundamentally removes this principal homeostatic mechanism. Once ventilation ceases, the internal physiological environment evolves as a closed system, in which oxygen progressively declines, carbon dioxide accumulates, and acid–base disturbances cannot be corrected actively until breathing resumes. Once a BH begins, $P_{aO_{2}}$ progressively declines and $P_{\mathrm{aC}O_{2}}$ rises as metabolism continues in the absence of pulmonary gas exchange. During extreme apnoea in elite BH divers, $P_{aO_{2}}$ may fall to ∼30 mmHg, while $P_{\mathrm{aC}O_{2}}$ may rise to ∼60 mmHg at the ‘breakpoint’ (Lindholm & Lundgren, 2006; Willie et al., 2015). The combined respiratory and metabolic acidosis progressively lowers pH, which cannot be corrected until ventilation resumes (Bain, Drvis et al., 2018; Ferretti, 2001; Lindholm & Lundgren, 2009). Initial O_2_ stores, $P_{\mathrm{aC}O_{2}}$, acid–base status and metabolic rate therefore establish the initial physiological trajectory of the BH, which is subsequently modified by cardiovascular, cerebrovascular and training‐related adaptations (Willie et al., 2015; Willie, Tzeng et al., 2014). Apnoeic tolerance consequently reflects the integrated influence of O_2_ storage and consumption, CO_2_ accumulation, buffering reserve, CDO_2_ and peripheral/central chemoreflex sensitivity.

Physiological benefits demonstrated while breathing hypoxia therefore cannot be assumed to translate directly to apnoea. Without ventilatory compensation, the magnitude, direction and functional consequences of changes in CO_2_, pH and tissue oxygenation may differ substantially. An intervention that alters metabolic or acid–base status before a BH might therefore exert effects distinct from those observed during altitude exposure or hypoxic exercise. This distinction is particularly relevant to KE supplementation, which acutely induces metabolic acidosis, hyperventilation and hypocapnia in free‐breathing conditions (Dearlove et al., 2021; Stubbs et al., 2017). Once apnoea begins, the hyperventilatory pathway underlying improved oxygenation is unavailable, whereas altered acid–base reserve, CO_2_ signalling and chemoreflex drive can persist. This physiological non‐equivalence provides the mechanistic framework for evaluating KE during BH diving and underpins the central hypothesis of this review (Figure 1).

## 3 ACID–BASE BALANCE: SETTING THE LIMITS OF APNOEA

Given that ventilation ceases during BH, CO_2_ cannot be eliminated and the progressive fall in pH cannot be attenuated through ventilatory compensation. Rising $P_{\mathrm{aC}O_{2}}$ and [H^+^] therefore become major determinants of chemoreflex‐mediated respiratory drive and apnoeic tolerance (Duffin, 2010). Buffering capacity depends on circulating bicarbonate, non‐bicarbonate buffers and tissue‐specific properties. The acid–base state at the onset of apnoea therefore establishes the initial buffering conditions that determine the subsequent rate of pH decline and the available buffering reserve. A diminished buffering reserve would accelerate acidosis, intensify chemoreflex activation and hasten the urge to breathe without necessarily altering the progression of hypoxaemia, thereby narrowing the functional safety margin between behavioural warning and physiological limit.

Pre‐apnoeic hyperventilation illustrates this principle. By lowering $P_{\mathrm{aC}O_{2}}$ and increasing blood and CSF pH, it delays the point at which CO_2_ and hydrogen‐ion accumulation generate an intolerable respiratory drive. When performed while breathing air, however, it results in only a modest increase in O_2_ stores and does not slow the continued decline in $P_{aO_{2}}$. Hypocapnia may therefore prolong BH duration by delaying respiratory discomfort while simultaneously weakening the warning signal that normally precedes hypoxic loss of consciousness.

KE ingestion creates a distinct pre‐apnoeic acid–base profile. Importantly, its effects should be distinguished mechanistically: KE ingestion induces ketosis and a metabolic acid load, whereas the associated hyperventilation and hypocapnia represent secondary compensatory responses that occur only while ventilation remains available. In free‐breathing conditions, this ventilatory response lowers $P_{\mathrm{aC}O_{2}}$ while increasing alveolar $P_{O_{2}}$ and $P_{aO_{2}}$. The magnitude and time course of these responses depend on the specific ketone formulation, dose and dosing regimen, making these factors important when extrapolating existing findings to potential pre‐apnoeic KE use. For example, during sustained hypoxic exposure, repeated ingestion of the ketone monoester (*R*)‐3‐hydroxybutyl (*R*)‐3‐hydroxybutyrate in 12.5 or 25 g boluses maintained circulating βHB concentrations at ∼3 mmol L^−1^. In parallel, capillary pH decreased from 7.421 at baseline to 7.395 after 1.5 h and 7.392 after 3 h, bicarbonate decreased from 26.0 to 22.7 and 21.0 mmol L^−1^, respectively, and capillary $P_{CO_{2}}$ fell from 40.7 to 37.6 and 33.2 mmHg, respectively (Stalmans et al., 2024). Although these pre‐apnoeic disturbances are smaller than the extreme hypercapnic–acidotic changes that can develop during prolonged apnoea, they are mechanistically important because they alter the acid–base starting point and reduce bicarbonate reserve before CO_2_ begins to accumulate. This combination of systemic acidosis and hypocapnia contrasts with the hypocapnic alkalosis produced by voluntary hyperventilation, creating a unique physiological state whose net effect on respiratory drive, safety margin and apnoeic tolerance cannot be predicted from existing evidence. Because CO_2_ readily crosses the blood–brain barrier, unlike circulating H^+^, KE‐induced hypocapnia may transiently alkalinise the CSF and attenuate central chemoreflex drive. In parallel, systemic acidosis may stimulate peripheral chemoreceptors, creating opposing effects on respiratory drive. KE ingestion also lowers circulating bicarbonate (Poffé et al., 2021; Stalmans et al., 2024), potentially limiting the buffering capacity once apnoea begins. Thus, a lower starting $P_{\mathrm{aC}O_{2}}$ might delay respiratory discomfort, whereas reduced bicarbonate reserve and blood acidosis might accelerate the subsequent fall in pH and augment peripheral chemoreflex activation.

βHB and AcAc also cross the blood–brain barrier through monocarboxylate transporters and can influence cerebral metabolism and the local acid–base environment (Pellerin et al., 2005). The integrated chemoreflex response will therefore reflect the balance among systemic acidosis, CSF alkalinisation, bicarbonate depletion and cerebral ketone uptake, ultimately determining how internal chemical signals are translated into the behavioural urge to breathe. The net effect of pre‐apnoeic KE ingestion on respiratory drive and BH tolerance remains unresolved.

KE‐induced acidosis can also modify systemic O_2_ transport through the Bohr effect. A fall in pH shifts the oxyhaemoglobin dissociation curve to the right, reducing haemoglobin–O_2_ affinity (Stalmans et al., 2024; Stalmans, Tominec, Lauriks et al., 2025; Stalmans, Tominec, Robberechts et al., 2025). In free‐breathing conditions, the accompanying hyperventilation and rise in alveolar $P_{O_{2}}$ might offset this reduction in affinity and preserve or even increase arterial oxygenation. During apnoea, however, O_2_ cannot be replenished, and alveolar and arterial $P_{O_{2}}$ progressively decline. A rightward shift might enhance peripheral O_2_ unloading and thereby support tissue O_2_ extraction, but it also lowers haemoglobin saturation at any given $P_{O_{2}}$. During apnoea, these effects can represent a physiological trade‐off rather than an inherently detrimental response, potentially facilitating O_2_ delivery to metabolically active tissues while altering the distribution and rate of utilisation of the finite O_2_ reserve. Whether this ultimately preserves or reduces the O_2_ remaining available to the brain during prolonged BH requires direct investigation.

## 4 DEFENDING THE BRAIN: CEREBRAL PERFUSION AND METABOLISM DURING APNOEA

Hypoxaemia and hypercapnia of apnoea are potent cerebrovascular dilators. Together with the accompanying rise in arterial and cerebral perfusion pressures, they increase cerebral blood flow (CBF) to defend CDO_2_ (Bain et al., 2015, 2016; Cross et al., 2013). This evolutionary conserved compensatory response preserves the cerebral metabolic rate for O_2_ (CMRO_2_) through most of a prolonged BH; only during the final ∼20% does CMRO_2_ decline by ∼25% (Bain et al., 2015, 2016, 2017; Bain, Ainslie et al., 2018). Severe hypercapnia, rather than hypoxia alone, is the principal driver of this late metabolic suppression (Bain et al., 2016, 2017; Bain, Ainslie et al., 2018; Bain, Drvis et al., 2018), consistent with reductions in CMRO_2_ observed during isolated hypercapnia during maintained ventilation (Caldwell et al., 2024).

Pre‐apnoeic hyperventilation lowers $P_{\mathrm{aC}O_{2}}$, causing cerebral vasoconstriction and reducing CBF and CDO_2_ before the BH begins. Although this may delay the subsequent rise in respiratory drive, it does not materially expand the finite O_2_ store available and may initiate apnoea from a lower cerebral perfusion state. Any performance benefit must therefore be weighed against this cerebrovascular cost. Likewise, KE ingestion modifies cerebral physiology through its ventilatory and acid–base effects. In free‐breathing conditions, KE‐induced hyperventilation raises $P_{aO_{2}}$ while lowering $P_{\mathrm{aC}O_{2}}$, resulting in a dose‐dependent, hypocapnia‐mediated reduction in resting CBF (Rourke et al., 2026). Despite the associated fall in CDO_2_, cerebral oxygenation increased during simulated exposure to 4000 m (Stalmans et al., 2024), and hypoxic alterations in neural signalling patterns were minimised at 3000 m (Vermaerke et al., 2026). This dissociation might reflect altered O_2_ extraction or utilisation but does not establish enhanced mitochondrial efficiency, cerebral O_2_ sparing or improved metabolic efficiency per se. Consistent findings in skeletal muscle demonstrate increased tissue oxygenation and O_2_ extraction without reduced O_2_ consumption (Stalmans et al., 2026). Any benefit during BH diving might therefore reflect altered O_2_ availability, distribution or extraction rather than reduced metabolic demand.

Caution is therefore required when extrapolating these findings to apnoea. Most KE‐related improvements in oxygenation occur while ventilation is maintained and are mediated, at least in part, by hyperventilation. Once the BH begins, this compensatory pathway is unavailable. Pre‐existing hypocapnia and reduced CBF may persist into the early phase of apnoea, but their duration and physiological relevance are uncertain because the progressive rise in $P_{\mathrm{aC}O_{2}}$ provides an increasingly powerful cerebrovascular dilatory stimulus as the BH continues. Thus, any KE‐induced reduction in pre‐apnoeic CBF should be considered a dynamic rather than a sustained cerebrovascular effect. Because freedivers already manipulate pre‐apnoeic ventilation, the incremental effect of KE might be limited or physiologically redundant. Ketone utilisation might, nevertheless, provide an alternative cerebral oxidative substrate independently of ventilation, particularly during progressive hypoxaemia, although whether this translates into greater energetic efficiency or meaningful O_2_ sparing during apnoea remains unknown. Whether this offsets reduced pre‐apnoeic CBF, altered acid–base balance and modified O_2_ unloading requires direct measurement of CBF, CDO_2_, O_2_ extraction and CMRO_2_ during KE‐supplemented apnoea. Until such data become available, any proposed benefit of KE during BH diving should be regarded as a physiological hypothesis rather than an established mechanism.

## 5 AUTONOMIC CONTROL: CONSERVING O_2_ DURING APNOEA

Autonomic regulation is central to BH performance. The diving response combines cardiac vagal activation, producing bradycardia, with sympathetic vasoconstriction that restricts peripheral perfusion and redistributes cardiac output towards the heart and brain (Ferretti, 2001; Heusser et al., 2009; Lindholm & Lundgren, 2009; Macefield et al., 2025). Together, these responses limit myocardial and peripheral O_2_ demand while defending central O_2_ delivery as hypoxaemia progresses.

This autonomic pattern evolves throughout apnoea in response to hypoxaemia, hypercapnia, chemoreflex activation and involuntary respiratory contractions. It cannot therefore be reduced to simple sympathetic or parasympathetic dominance, nor inferred reliably from isolated heart‐rate variability (HRV) indices (Billman, 2013). Effective performance instead depends on the coordinated regulation of heart rate (HR), peripheral vascular resistance and arterial pressure.

Acute KE ingestion during hypoxic exposure has been associated with higher HR and lower HRV (Stalmans et al., 2024; Stalmans, Tominec, Robberechts et al., 2025). These changes might reflect peripheral chemoreceptor activation by metabolic acidosis, reflex effects of hyperventilation and hypocapnia, or direct ketone‐mediated modulation of autonomic signalling (Kimura et al., 2011). However, changes in HR and HRV alone cannot distinguish reduced cardiac vagal modulation from increased sympathetic activity and therefore do not establish sympathetic activation directly. A higher pre‐apnoeic HR could increase myocardial O_2_ demand, whereas augmented vasoconstriction might either support central blood redistribution or increase cardiovascular strain. Reduced vagal modulation could also attenuate the O_2_‐conserving bradycardia. The net effect is therefore uncertain and likely to depend on the KE dose, acid–base disturbance, training status and BH discipline. Direct assessment during KE‐supplemented apnoea is required to establish its consequences for performance and safety.

## 6 TRAINING ADAPTATION: ACUTE VERSUS REPEATED KETOSIS

The physiological response to apnoea is highly trainable. Elite freedivers develop a recalibrated relationship among chemical stimuli (CO_2_, pH and O_2_), respiratory discomfort and the behavioural decision to breathe. Training is associated with reduced ventilatory sensitivity to CO_2_, allowing greater hypercapnic tolerance and longer BH duration without abolishing chemoreflex signalling (Bain, Ainslie et al., 2018; Bain, Drvis et al., 2018). It might also strengthen cortical control over respiratory drive, enabling divers to delay the behavioural response to mounting respiratory discomfort without altering the progression of hypoxaemia.

In‐water performance also reflects ‘aquaticity’, which is the integrated expression of biomechanical efficiency, neuromuscular control, energetic regulation and perceptual–mental stability under aquatic constraints (Drviš et al., 2026). These factors influence movement economy and metabolic demand and thereby modify the rates of O_2_ depletion and CO_2_ accumulation during BH diving. Against this adapted physiological background, the effects of KE may depend critically on exposure timing. Acute ingestion induces transient ketosis, metabolic acidosis, hyperventilation and hypocapnia, accompanied by a dose‐dependent reduction in CBF (Rourke et al., 2026; Stalmans et al., 2024). In contrast, 14 days of daily KE supplementation was associated with increased CBF when assessed in the fasted, non‐ketotic state following the intervention in adults with obesity (Stalmans & Poffé, 2026; Walsh et al., 2021). Importantly, these observations reflect different physiological states and populations and should not be interpreted as evidence that repeated KE exposure reverses the acute cerebrovascular response. Rather, they raise the possibility that repeated supplementation might induce adaptations that persist beyond the period of acute ketosis, although their mechanisms and relevance to freedivers remain unknown. Acute KE ingestion has also been associated with higher HR and lower HRV, whereas repeated supplementation might attenuate overreaching‐associated sympathetic overactivity (Stalmans et al., 2024; Stalmans, Tominec, Robberechts et al., 2025). Moreover, KE intake increases circulating levels of erythropoietin, magnifies angiogenic signalling and stimulates capillarisation, while increasing microvascular function and elevating biomarkers for vessel integrity (Poffé et al., 2021), potentially enhancing apnoeic oxygen delivery.

Acute pre‐dive use and repeated supplementation during training should therefore not be conflated. Recurrent 3–5 h episodes of KE‐induced hypocapnia could theoretically influence the adaptations promoted by hypercapnic BH training, although this hypothesis remains untested. Longitudinal, training‐specific studies are required to determine whether repeated ketosis supports or disrupts freediving adaptation.

## 7 STATIC VERSUS DYNAMIC APNOEA: DISCIPLINE‐SPECIFIC CONSTRAINTS AND IMPLICATIONS

Static apnoea (SA) and dynamic apnoea (DA) impose contrasting physiological demands. SA is performed with minimal voluntary movement and muscle activation, with performance determined largely by metabolic economy, tolerance to rising CO_2_ and the ability to suppress the urge to breathe (Bain, Drvis et al., 2018; Schagatay, 2009). In contrast, DA requires sustained locomotion and therefore imposes greater metabolic, neuromuscular and coordinative demands (Drviš et al., 2025). Systemic O_2_ consumption is substantially higher, making movement efficiency, propulsion cost and the strategic allocation of finite energetic resources major performance constraints, features encompassed by energetic aquaticity (Drviš et al., 2026).

These discipline‐specific demands are likely to modify the effects of KE, because the relative importance of acid–base disturbance, respiratory drive and finite O_2_ stores is likely to be greater in SA, whereas substrate utilisation, locomotor efficiency and exercising‐muscle O_2_ demand become increasingly relevant in DA. In SA, where metabolic demand is low and physiological stability critical, even modest disturbances in acid–base balance or chemoreflex signalling can disproportionately reduce apnoea tolerance without altering the progression of hypoxaemia (Bain, Drvis et al., 2018; Schagatay, 2009). KE‐induced acidosis and bicarbonate depletion might therefore be especially detrimental. In DA, greater muscular work increases the potential relevance of substrate utilisation and bioenergetic efficiency; however, ketone oxidation appears limited when alternative substrates, particularly glucose, are available (Dearlove et al., 2021; Petrick et al., 2020), and KE ingestion has not been shown to reduce skeletal muscle O_2_ consumption (Stalmans et al., 2026) and even elevates whole‐body oxygen consumption rate, potentially owing to increased respiratory effort (Poffé et al., 2021; Stalmans et al., 2024). Any putative metabolic benefit must therefore be weighed against potential disturbances in acid–base status, bicarbonate reserve, neuromuscular control and respiratory drive, whose relative importance is likely to differ between SA and DA. Accordingly, KE should be evaluated separately in each discipline, because failure to account for their distinct physiology might obscure genuine effects or misrepresent ergogenic potential.

## 8 SAFETY AND PERCEPTION: PROTECTING THE WARNING SIGNAL

During apnoea, rising CO_2_ and falling pH drive the urge to breathe, whereas severe hypoxaemia imposes the ultimate physiological limit and can precipitate loss of consciousness (Ferretti, 2001; Lindholm & Lundgren, 2009). Because respiratory discomfort normally precedes critical cerebral hypoxia, this temporal separation creates a crucial safety margin. Any intervention that delays respiratory discomfort without slowing O_2_ depletion can narrow this safety margin.

Pre‐apnoeic hyperventilation illustrates the risk. Hypocapnia delays chemoreflex‐mediated respiratory drive but does not prevent the progressive fall in $P_{aO_{2}}$, thereby increasing the likelihood of hypoxic loss of consciousness (Ferretti, 2001; Lindholm & Lundgren, 2009). Previous metabolic and nutritional manipulations provide relevant proof of principle that prolonging BH duration does not necessarily increase physiological safety. Fasting has been shown to prolong static apnoea while allowing BHs to terminate at lower O_2_ levels, an effect associated with altered CO_2_ accumulation (Elia et al., 2022; Schagatay & Lodin‐Sundström, 2014). Moreover, when fasting was combined with pre‐apnoeic hyperventilation, BH duration increased further while severe hypoxaemia became more frequent during repeated maximal apnoeas (Elia et al., 2024). These findings demonstrate that interventions modifying the pre‐apnoeic metabolic or respiratory state can alter the relationship between performance, internal warning signals and the physiological limit.

Nutritional interventions that modify redox and nitric oxide biology provide a related example. Extreme apnoea exposes the brain to marked hypoxaemic–hypercapnic stress and has been shown to increase cerebral oxidative–nitrosative stress, with implications for cerebral bioenergetic function (Bailey et al., 2024). Vitamin C supplementation has also been shown to modify neutrophil antioxidant defences, inducible nitric oxide synthase expression and nitric oxide‐related responses during diving apnoea (Sureda et al., 2004). Although these findings do not establish an ergogenic effect, they demonstrate that antioxidant supplementation can alter redox homeostasis and nitric oxide‐linked responses to apnoeic stress. This is important because reactive oxygen and nitrogen species are not simply biomarkers of oxidative–inflammatory–nitrosative injury; they also contribute to vascular signalling, mitochondrial regulation and hormetic adaptation to hypoxia–reoxygenation. Nutritional strategies that blunt or redirect redox signalling can therefore influence apnoeic physiology through effects on endothelial function, CBF, peripheral vasoconstriction, O_2_ utilisation and recovery, even when effects on BH duration remain uncertain.

Dietary nitrate studies provide further context. Nitrate supplementation has been reported to improve arterial O_2_ saturation following dynamic apnoea (Patrician & Schagatay, 2017), whereas acute beetroot juice supplementation did not significantly alter peripheral O_2_ saturation, HR or pulmonary gas‐exchange responses during submaximal static or dynamic apnoea (Barlow et al., 2018). Together, these studies show that nutritional interventions should be evaluated not only by BH duration, but also by their effects on redox balance, nitric oxide bioavailability, vascular control, chemoreflex signalling and the safety margin before hypoxic loss of consciousness.

Likewise, KE could alter CO_2_ signalling, chemoreflex drive or the respiratory perception. This does not establish that KE is inherently hazardous; rather, it raises the possibility that physiological warning signals could become dissociated from the progression of hypoxaemia. No study has directly assessed performance or safety outcomes during KE‐supplemented BH diving; consequently, any proposed effect of KE on the safety margin between respiratory warning signals and hypoxic loss of consciousness remains hypothetical and should not presently be interpreted as evidence of either harm or protection.

## 9 RESEARCH PRIORITIES: TESTING KETONE ESTER DURING APNOEA

No study has directly examined exogenous ketosis during BH diving, precluding evidence‐based recommendations for performance or safety. Findings from breathing hypoxia and exercise cannot be extrapolated confidently because they do not reproduce the absence of ventilation or the progressive hypoxaemia, hypercapnia and acidosis of apnoea. Randomised, double‐blind, placebo‐controlled studies should determine how KE affects the principal limits of BH tolerance, including arterial and cerebral acid–base status, $P_{aO_{2}}$ and $P_{\mathrm{aC}O_{2}}$ kinetics, bicarbonate reserve, CBF, CDO_2_, O_2_ extraction, CMRO_2_ and the Hb–O_2_ dissociation curve. Concurrent assessment of central and peripheral chemoreflex sensitivity, respiratory discomfort, involuntary respiratory contractions and the behavioural breakpoint should establish whether KE alters warning signals independently of O_2_ depletion. Cardiovascular measurements should also quantify bradycardia, peripheral vasoconstriction, arterial pressure regulation and cerebral perfusion. To ensure appropriate interpretation, designs must distinguish SA from DA, trained from untrained participants, acute from repeated supplementation, and KE from other ketone formulations.

## 10 CONCLUSION

KE supplementation might improve oxygenation during breathing hypoxia, but apnoea removes the ventilatory mechanism underlying much of this benefit. Persistent effects on acid–base balance, bicarbonate reserve, CO_2_ signalling, CBF and autonomic control could, nevertheless, alter BH performance and safety in unpredictable ways. Any gain in O_2_ delivery, extraction or ketone‐supported bioenergetics must be weighed against accelerated acidosis, altered chemoreflex drive and potential narrowing of the safety margin between behavioural warning and hypoxic loss of consciousness. Exogenous ketosis should therefore be regarded not as an established ergogenic aid for freediving, but as an untested strategy whose effects might prove beneficial, neutral or detrimental depending on the balance among these competing mechanisms and whose efficacy and safety require direct evaluation during apnoea.

## AUTHOR CONTRIBUTIONS

Ivan Drviš and Željko Dujić conceived the idea and wrote the first draft of the manuscript with input from Myrthe Stalmans, Dario Vrdoljak, Nikola Foretic and Damian M. Bailey. All authors approved the final version submitted for publication and agree to be accountable for all aspects of the work in ensuring that questions related to the accuracy or integrity of any part of the work are appropriately investigated and resolved. All persons designated as authors qualify for authorship, and all those who qualify for authorship are listed.

## CONFLICT OF INTEREST

Damian M. Bailey is Editor‐in‐Chief of *Experimental Physiology* and outgoing Chair of the Life Sciences Working Group and outgoing member of the Human Spaceflight and Exploration Science Advisory Committee to ESA. Damian M. Bailey is a current member of the European Space Agency‐Human and Robotic Exploration‐Biology Panel and Space Exploration Advisory Committees to the UK and Swedish National Space Agencies.

## GENERATIVE AI STATEMENT

ChatGPT (OpenAI) was used to assist with the conceptual drafting of Figure 1. All content was critically reviewed, revised and approved by the authors, who take full responsibility for the manuscript.
